# Identification of Protective Pneumococcal T_H_17 Antigens from the Soluble Fraction of a Killed Whole Cell Vaccine

**DOI:** 10.1371/journal.pone.0043445

**Published:** 2012-08-14

**Authors:** Kristin L. Moffitt, Richard Malley, Ying-Jie Lu

**Affiliations:** Division of Infectious Diseases, Boston Children's Hospital, Harvard Medical School, Boston, Massachusetts, United States of America; Los Angeles Biomedical Research Institute, United States of America

## Abstract

Mucosal or parenteral immunization with a killed unencapsulated pneumococcal whole cell antigen (WCA) with an adjuvant protects mice from colonization by a T_H_17 CD4+ cell-mediated mechanism. Using preparative SDS gels, we separated the soluble proteins that compose the WCA in order to identify fractions that were immunogenic and protective. We screened these fractions for their ability to stimulate IL-17A secretion from splenocytes obtained from mice immunized with WCA and adjuvant. We identified 12 proteins within the stimulatory fractions by mass spectrometry; these proteins were then cloned, recombinantly expressed and purified using an *Escherichia coli* expression system. The ability of these proteins to induce IL-17A secretion was then evaluated by stimulation of mouse splenocytes. Of the four most stimulatory proteins, three were protective in a mouse pneumococcal serotype 6B colonization model. This work thus describes a method for identifying immunogenic proteins from the soluble fraction of pneumococcus and shows that several of the proteins identified protect mice from colonization when used as mucosal vaccines. We propose that, by providing protection against pneumococcal colonization, one or more of these proteins may serve as components of a multivalent pneumococcal vaccine.

## Introduction

While pneumococcal conjugate vaccines (PCVs) have resulted in significant reductions in rates of invasive pneumococcal disease due to vaccine-serotypes, infection with *Streptococcus pneumoniae* remains an important public health issue, particularly in developing countries [1]. Post-PCV-licensure surveillance of pneumococcal disease also demonstrates replacement of carriage and invasive disease serotypes with those not covered in the PCVs [2],[3],[4]. Additionally, the impact of PCVs on mucosal disease such as otitis media and pneumonia has been less clear. For these reasons and with the goal of developing a vaccine with lower cost of goods and complexity of manufacture than PCVs, alternative approaches to pneumococcal vaccination are a high global health priority.

Many strategies have been proposed and evaluated (reviewed in [5]). One approach that is being actively pursued and is currently in a Phase I trial involves the use of killed whole pneumococcal cells. Our group has shown that either mucosal or parenteral immunization with a killed unencapsulated pneumococcal whole cell vaccine (denoted WCV when administered with appropriate adjuvant) protects mice against pneumococcal colonization with a clinical isolate of serotype 6B and fatal aspiration-sepsis with isolates of serotypes 3 and 5 [6], [7], [8]. Perhaps due to the unencapsulated nature of the immunogen, and in light of recent studies that reveal robust immunological responses directed against pneumococci of diverse genotypes and serotypes [9], immunization with WCV is expected to confer protection against a wide variety of strains. Protection against aspiration/sepsis is transferrable by passive transfer of sera from immunized rabbits to mice, whereas depletion of CD4+ T cells in immunized mice at the time of challenge results in no detectable loss of protection, pointing to a critical role of antibodies [7]. In contrast immunity to colonization conferred by immunization with WCV is dependent on CD4+ T cells and IL-17A [7]. Indeed, in the mouse model used, congenital absence of antibodies, IFN-γ or IL-4 has no effect, whereas depletion of CD4+ T cells or congenital absence of the IL-17A receptor is associated with loss of protection against pneumococcal colonization [10], [11].

As methods for formulating the WCV preparation have been optimized for GMP-grade and scale production, various modes of inactivation of the parent WCV strain, RM200 (a strain derived from the unencapsulated strain Rx1, in which the autolysin A gene has been disrupted and the pneumolysin gene has been replaced with one encoding for a pneumolysoid PdT [7]), have been investigated. We have shown that inactivation methods using beta-propiolactone or organic solvents, such as chloroform, that retain the soluble proteins of RM200 (by avoiding the requirement for post-inactivation washing in the original ethanol-inactivated WCA [6]) produce significantly more potent vaccine preparations, such that 100 times lower WCA concentration confers the same immune and protective responses as compared to a WCA preparation in which soluble proteins have been removed [8]. These data led us to explore the hypothesis that proteins with protective potential may be present in the soluble fraction of the WCA (released during growth or by inactivation): consistent with this hypothesis, we also showed that intranasal immunization with the soluble fraction of chloroform-inactivated WCA (thereby lacking the pelleted whole cells and their contents) conferred highly significant reductions in colonization compared with adjuvant-alone immunized mice [8].

The identification of the protective components in the soluble fraction of the WCA has at least two important advantages. First, by its nature, the WCA is a highly complex preparation, consisting of a mixture of antigens among over 2000 presumed expressed pneumococcal proteins. For manufacturing and quality control issues, knowledge of components of the vaccine that are particularly relevant to protection would be very useful. Lot-to-lot variability could be examined by determining the concentration of individual proteins among the different lots of WCA. Secondly, currently there are several efforts aimed at the development of novel protein-based pneumococcal protein vaccines. The success of the pneumococcal conjugate vaccine against invasive disease can be ascribed (at least in part) to the remarkable impact of these vaccines on colonization: by CDC estimates, over 2/3 of cases prevented by pneumococcal conjugate vaccine is the result of indirect, or herd, protection [12]. For this reason, ideally, an alternative strategy should confer protection against both pneumococcal invasive disease and colonization and thus the inclusion of antigens that target pneumococcal colonization in a protein-based vaccine would be advisable. There are many examples of pneumococcal proteins that have been investigated as components of a protein subunit vaccine [5], and combinations of several of those explored have been shown to significantly reduce colonization in mice in a CD4 + T cell- [13] and IL-17A- [14] dependent manner when used as intranasal immunogens adjuvanted with cholera toxin. This work aimed to identify the immunogenic and protective proteins in the soluble fraction of an inactivated WCA preparation and evaluate the potential of these antigens to reduce colonization when used as recombinant protein vaccines.

## Materials and Methods

### Bacterial strains

The whole cell pneumococcal vaccine was prepared from strain RM200 that is derived from a parent Rx1 strain that is autolysin-negative, capsule-negative, and expresses a non-hemolytic pneumolysoid variant in the place of the hemolytic pneumolysin [8]. For this work, RM200 was grown to an OD*_600_* of 1.0 (corresponding to approximately 6.0×10^8^ CFU/ml), washed in Lactated Ringer's solution supplemented with 10% sucrose, concentrated to an OD_600_ of 32, and inactivated by mixing with chloroform as previously described (1/40 [chloroform vol/bacterial cell vol] for 2 hours) [8]. Chloroform-inactivated whole cell antigen (hereafter referred to as WCC) was not washed; any residual organic solvent was sublimated away from the preparation during lyophilization. WCC supernatant was harvested by centrifuging WCC rehydrated in sterile water at 16,000×*g* for 5 minutes and collecting the aqueous layer separate from the pellet. Protein estimation was performed using Total Protein Kit with bovine serum albumin as a standard (Pierce).

### Animal models and ethics statement

For all experiments, 4–6 week old C57BL/6J mice from Jackson Laboratories (Bar Harbor, ME) were used. All immunizations were performed by instilling 20 μl of a mixture of PBS, 1 μg of cholera toxin (CT; List Biologicals, Campbell, CA) and either 4 μg of the indicated purified pneumococcal protein or 100 μg of WCC as immunogens onto the nares of gently restrained unanesthetized mice. Control animals were immunized with CT mixed in PBS alone to control for any nonspecific effect of the adjuvant alone. Animals were immunized intranasally twice at one-week intervals. Spleens were harvested from immunized animals 2–4 weeks following last immunization (without pneumococcal challenge) for stimulations with WCC supernatant fractions and purified pneumococcal proteins. Immunized animals that were subsequently challenged received an inoculum consisting of 10^7^ CFU of a clinical type 6B pneumococcal strain (0603; described in [6]) in 10 μl volume intranasally while awake. These mice were euthanized by CO2 inhalation one week following challenge; tracheal washes were obtained and assessed for density of pneumococcal colonization as previously described [6]. All animal work was performed in accordance with NIH guidelines and approved by the IACUC of Children's Hospital Boston and Harvard Medical School.

### Molecular mass separation of WCC supernatant fractions

WCC supernatant (containing 800–900 μg of protein) was mixed with LDS 4x sample buffer and loaded into the 8 center lanes of a NuPAGE 4–12% Bis-Tris 12-lane gel (Invitrogen Corporation, Carlsbad, CA) then resolved by sodium dodecyl sulfate-polyacrylamide gel electrophoresis (SDS-PAGE) for 30–40 minutes at 200V. The preparative gel was then equilibrated in elution buffer (2 mM phosphate buffer at pH 7.0) for three 20-minute equilibrations. Transverse elution of the proteins through the thickness of the preparative gel was performed at constant 300V for forty minutes (current ranged from 65–90 mAmp) in elution buffer in a mini whole gel transverse eluter system (Bio-Rad Laboratories, Hercules, CA). Current was reversed for 15 seconds at the end of the elution and individual fractions were collected in fourteen chambers beneath the preparative gel and harvested into separate tubes. The protein content of each fraction was quantified by Total Protein Kit (Biorad).

### Mass spectrometry

After determining which fractions were most immunogenic in the IL-17A splenocyte stimulation assays described below, MS identification of the proteins contained in the bands comprising the most immunogenic fractions was carried out at the Taplin Biological Mass Spectrometry Facility of Harvard Medical School, Boston, MA.

### Protein expression and purification

Gene sequences for twelve of the proteins identified from immunogenic fractions were amplified by PCR using primers designed from the sequence published for serotype 4 strain TIGR4 (Table 1). Two restriction enzyme sites, BamHI and SphI, were included in primers to allow cloning into the expression vector pQE30 (Qiagen, Inc) which also incorporates a 6x-histidine (*his*) tag. PCR products were ligated into the pQE30 expression vector and ligation products were transformed into competent *Escherichia coli* (XL/Blue) cells. Transformed cells were selected by growth on LB ampicillin and the identity of the insert confirmed by sequencing. Successfully transformed *E. coli* cells were grown in 1L LB ampicillin and protein expression was induced with 0.1 mM IPTG overnight at room temperature. Pelleted *E. coli* were lysed in lysis buffer (20 mM Tris-HCl, 500 mM NaCl with lysozyme at 1 mg/ml and DNAse at 10 μg/ml) and sonicated. Protein was purified over a column of Ni-NTA-agarose beads that had been equilibrated with lysis buffer. The protein-bound column was washed with lysis buffer/40 mM imidazole, and proteins were eluted in 250 mM imidazole. Proteins were run on SDS-PAGE under reducing conditions and gels were Coomassie-stained to confirm protein expression and purity. Purified proteins were desalted on a PD-10 Sephadex column using PBS prior to use in cellular stimulation assays.

**Table 1:** T_H_17 cell antigens isolated from immunogenic fractions of soluble portion of RM200, predicted function, and primers used for expression in *E. coli.*

Locus in TIGR4	Predicted function	Primer sequence (5′–3′) using Bam HI and SphI restriction sites
SP 0435	Elongation factor	up- GCGGATCCATGATTGAAGCAAGTAAAT down- GCGCATGCTTAGGCACGAGAAACGTAAG
SP 0516	Heat shock protein	up- GCGGATCCATGGCCCAAGATATAAAAAATGAAG down- GCGCATGCTTAGTTATACACCACTACCATT
SP 0862	Ribosomal protein S1	up- GCGGATCCATGAACGAATTTGAAGATTTGC down- GCGCATGCTTAAAGTTCGATATCACCAAACAAATC
SP 0946	Hypothetical	up- GCGGATCCATGAATACAAATCTTGCAAG down- GCGCATGCCTAAATCAACTCTGTCCC
SP 1297	Flavodoxin	up- GCGGATCCATGGCATTAGCAAAAATTGTATTTG down- GCGCATGCTTATCCTACTTTAGCAGCCAATTC
SP 1415	Glucosamine-6-phosphpate isomerase	up- GCGGATCCATGAAAGTTATTAAAGTTGAAAAC down- GCGCATGCTTAGAGTAAGCTAAGCGCTTCAGC
SP 1458	Thioredoxin reductase	up- GCGGATCCATGTACGATACTATTATTATCGG down- GCGCATGCTTAACTATGTTCTGTAATGAATT
SP 1534	Putative manganese-dependent inorganic pyrophosphatase	up- GCGGATCCATGTCCAAGATTCTAGTATTTG down- GCGCATGCTTACGCATTAAAGCTTTCAGTC
SP 1572	Non-heme iron containing ferritin	up- GCGGATCCATGAATGAGGTAAAGAAAATGG down- GCGCATGCTTACAAACCAGGTGCTTGTCCAAG
SP 1733	Putative phosphatase	up- GCGGATCCATGGAAATTTCATTATTAACAGATG down- GCGCATGCTCATTCTGCATCCTCCTCGTTC
SP 2070	Glucose-6-phosphate isomerase	up- GCGGATCC ATGTCACATATTAAATTTGATTATTC down- GCGCATGCTTATAGACGTGCGTTAAGTTCTTTG
SP 2092	UTP-glucose-1-phosphate uridyltransferase	up- GCGGATCC ATGACATCAAAAGTTAGAAAGGC down- GCGCATGCTTATTCCTTCTCAGTCAATTCTTTTC

### Splenocyte stimulations and ELISAs

Spleens were harvested from WCC-immunized C57BL/6 mice and were processed as previously described into a cellular suspension [10] and stimulated either with 1) equivalent concentrations of size-separated fractions of WCC supernatant or with 2) 10 μg/ml of recombinant protein. Splenocytes were incubated for 3 days; plates were then spun to pellet cells, after which the supernatants were collected and assayed for IL-17A using a mouse IL-17A ELISA kit (R&D Systems, Inc).

### Statistical analysis

All statistical analyses were carried out using PRISM (version 4.0; GraphPad Software Inc). Colonization densities and IL-17A values among immunization groups were compared by Mann-Whitney U test or by the Kruskal-Wallis test with Dunn's correction for multiple comparisons. P values <0.05 were considered to represent statistical significance.

## Results

### Identification of stimulatory fractions by preparative SDS PAGE

**Figure 1:**
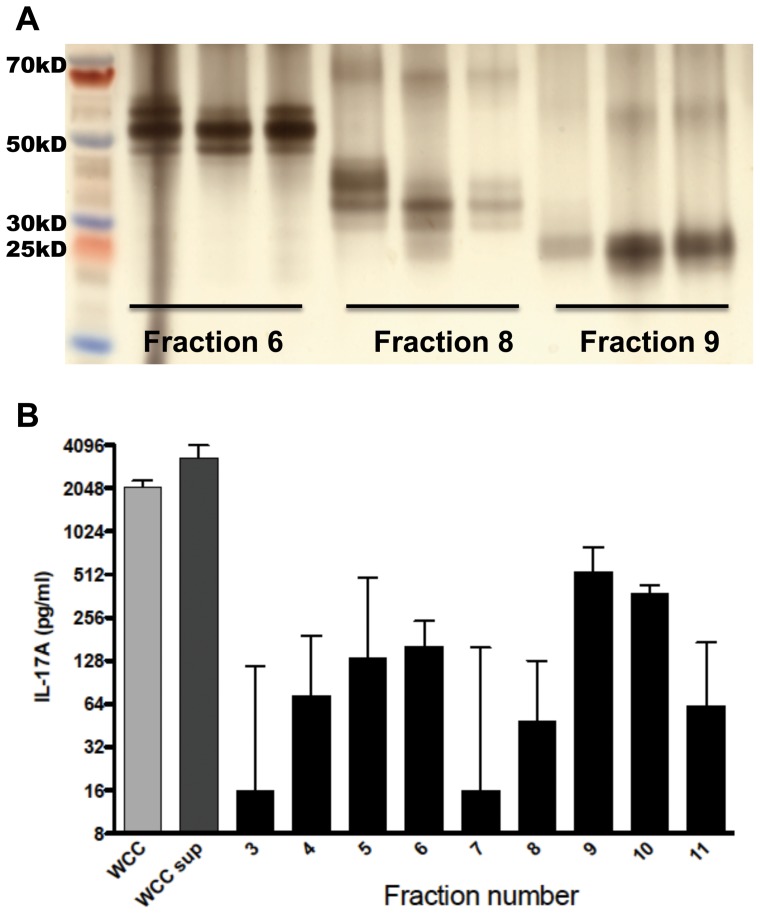
Size separation of fractions and stimulation of splenocytes. A. SDS-PAGE of fractions was performed and gel was silver-stained; shown here are the electrophoretic patterns of three fractions eluted into the same chamber during different transverse elutions. B. Results from stimulation of splenocytes from WCC immunized mice (n = 6) with equal concentrations of each fraction. Supernatants were collected after 3 days of incubation and IL-17A concentration in the supernatant was measured by ELISA. IL-17A values are shown here, normalized to the DMEM-stimulated response of each animal. Bars represent medians with interquartile range. WCC: chloroform-inactivated pneumococcal whole cell antigen, 10 µg protein/ml; WCCsup: soluble fraction of WCC, 7 µg protein/ml.

While the supernatant of WCC (WCCsup) contains approximately 15% of the total protein of freshly rehydrated noncentrifuged WCC, prior work has demonstrated that, with respect to protection against pneumococcal colonization, retention of the soluble portion of the vaccine yielded a 100-fold more potent whole cell preparation when compared with an ethanol-inactivated preparation in which the soluble fraction was removed by serial washings of the pellet [8]. In order to identify immunogenic proteins within WCCsup, we proceeded to fractionate WCCsup proteins by preparative SDS PAGE. After proteins were separated by SDS PAGE, they were eluted from the gel by transverse elution with recovery of approximately 50% of the loaded protein. Fourteen fractions were generated from each transverse elution. By evaluating SDS-PAGE of collected fractions, we confirmed consistency of elutions across different experiments, as shown by the similarly sized bands in fractions from elutions performed at different times (Figure 1A). This allowed us to combine fractions from several elutions to yield approximately 3 ml of each fraction with concentrations ranging from 27–43 μg/ml. By SDS-PAGE, it appeared that each fraction consisted of 2–4 predominant bands.

Fractions were all brought to the same protein concentration and used to stimulate splenocyte suspensions obtained from WCC-immunized mice. The WCC and WCCsup elicited median IL-17A responses of 2100 and 3300 pg/ml respectively (Figure 1B). The most stimulatory fractions were fractions 9 and 10, which elicited median IL-17A values of 530 and 380 pg/ml, respectively. These two fractions and, as a control that would allow us to exclude noncontributory proteins, one non-stimulatory fraction were re-applied onto SDS-PAGE and the most predominant bands within each fraction were submitted for mass spectrometry analysis.

### Bioinformatic targeting of proteins identified by mass spectrometry

Peptides within the submitted bands were identified and matched to the proteomic sequence of pneumococcal strain TIGR4. Proteins with more than 2 identified peptide matches were considered positively identified. Using these methods, 37 proteins were identified in the stimulatory fractions but not in the non-stimulatory counterpart. The corresponding sequences of identified proteins were analyzed bioinformatically; we prioritized antigens that were highly conserved (>85% amino acid homology) among the published pneumococcal sequences and with <10% homology to human proteins. This narrowed the panel of proteins to 12 proteins, which we examined in more detail; these are listed in Table 1.

### Several recombinant proteins elicit IL-17A from splenocytes

**Figure 2:**
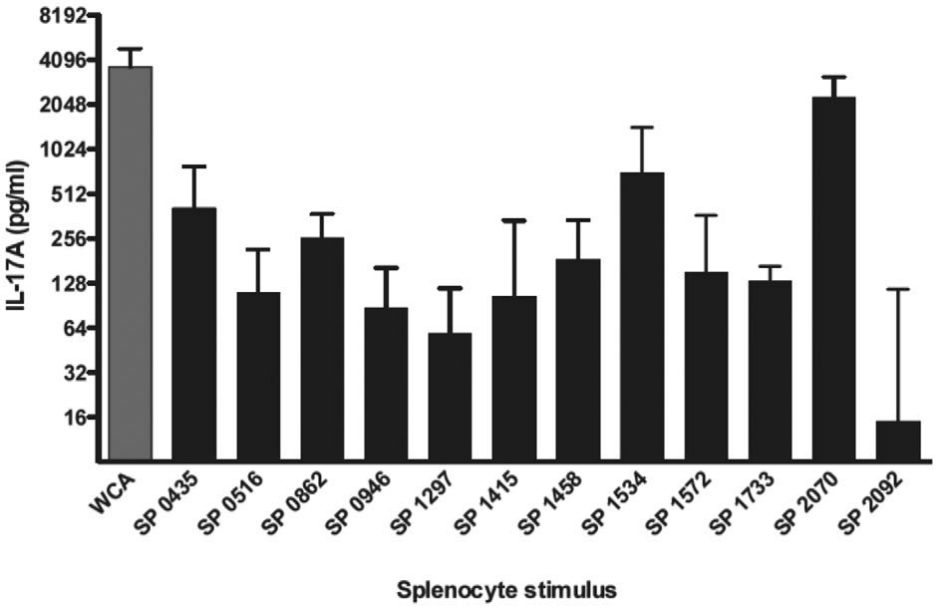
Stimulation of splenocytes from immune mice with purified recombinant proteins. Mice (n = 7–10) were intranasally immunized with WCC and cholera toxin as described. Splenocytes from immunized mice were stimulated with 10 μg/ml of the indicated recombinant protein for 3 days, after which supernatants were harvested and assayed for IL-17A concentration. Values are normalized to the DMEM stimulated response for each animal. Bars represent medians with interquartile ranges.

The 12 identified proteins were successfully expressed in *E. coli* and purified. Gels for each of the 12 proteins demonstrated predominant size-appropriate single bands suggesting successful purification. Each recombinant protein was used at 10 μg/ml to stimulate splenocytes from WCC-immunized mice. Several individual proteins elicited robust median IL-17A responses; in particular, SP2070, SP1534, SP0435 and SP0862 elicited the highest median IL-17A responses (range 260–2300 pg/ml) as shown in Figure 2. Other recombinant proteins, such as SP2092, were clearly less stimulatory, suggesting that not all proteins contained within bands from stimulatory fractions were contributing to the IL-17A response.

### Proteins administered intranasally with cholera toxin reduce nasopharyngeal carriage

**Figure 3:**
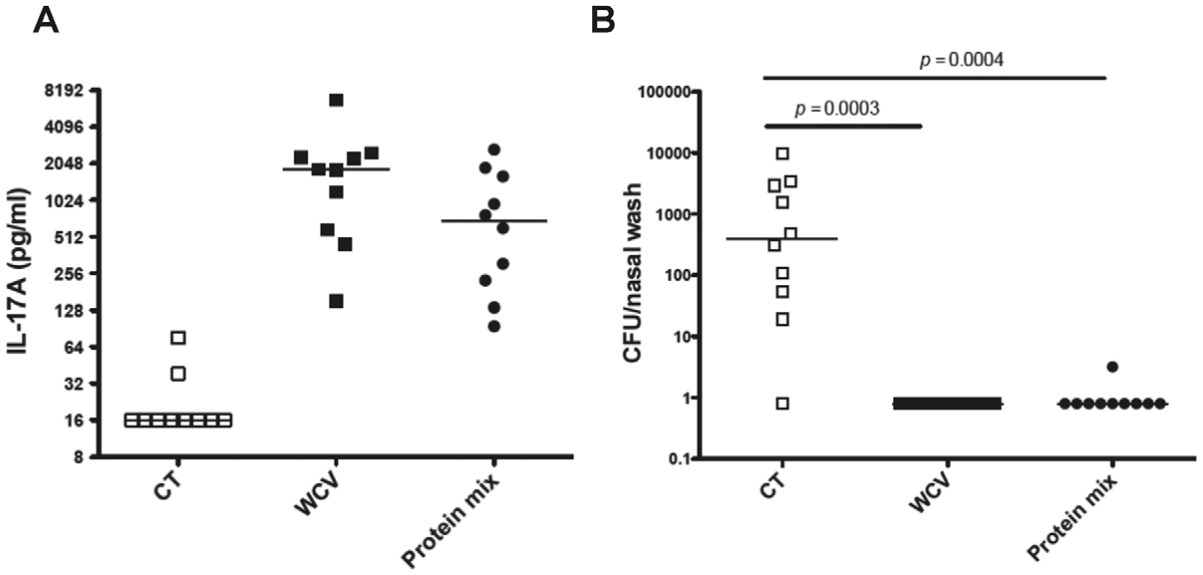
Protection against colonization by intranasal immunization with a mixture of proteins. Mice were intranasally immunized with a mixture (Protein mix) containing 4 μg of SP0435, SP1534, and SP2070 and 1 μg of cholera toxin (CT) as adjuvant; control mice received CT alone or WCC with CT (WCV). Blood was obtained 3 weeks after last immunization and mice were challenged one week later with strain 0603. Density of colonization was determined one week after challenge by quantifying pneumococcal carriage from nasal washes. (A) IL-17A production was determined *in vitro* from whole blood stimulated with pneumococcal whole-cell antigen. (B) Mice immunized with the WCV or the protein mixture were significantly protected against colonization compared to mice that received CT alone. Bars indicate median values and P values were determined by Mann-Whitney U test compared to mice immunized with CT alone.

To assess the protective efficacy of recombinant proteins, we began with a preparation containing a mixture of the three antigens that were most stimulatory in the splenocyte stimulations (Figure 2) and compared the ability of this mixture vs. WCC to elicit a T_H_17 response and protect against colonization. The T_H_17 responses following immunization with the mixture of proteins or WCV were similar and in both cases significantly higher compared to that of mice immunized with CT alone (*p* = 0.0001 for both, Figure 3A). Our experience with the WCV administered intranasally indicates that a post-immunization IL-17A value of ≥250 pg/ml elicited following whole blood stimulation with WCA is a good predictor of protection against colonization [10]. Accordingly, animals immunized with the combination vaccine were significantly protected from colonization (over 2 log reduction in median density of colonization, *p* = 0.0004 compared with CT immunized animals), and the combination vaccine was as protective as the WCV in this experiment, with all but one nasal wash being free of detectable pneumococci (Figure 3B).

**Figure 4:**
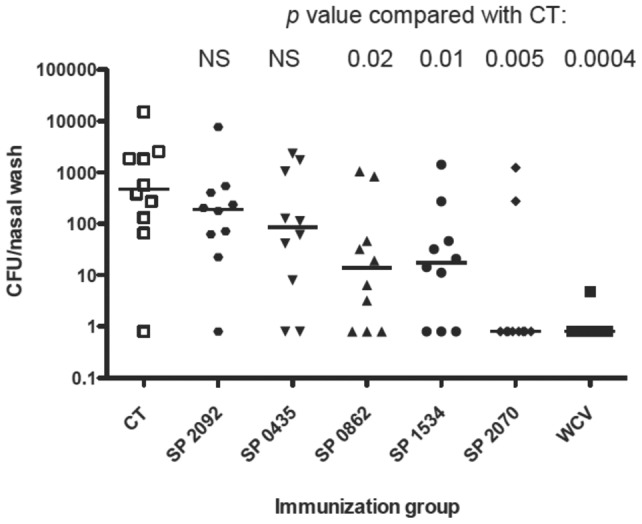
Immunization with individual proteins confers protection against nasopharyngeal carriage. Mice were intranasally immunized twice with 1 µg of cholera toxin alone (CT) or CT combined with the proteins as indicated. Four weeks after the second immunization, mice were intranasally challenged with strain 0603; density of colonization was determined one week later. Bars indicate median values and nasal colonization density was compared by the Mann-Whitney *U* test.

Having shown that a combination of these proteins protected animals from colonization, we performed immunizations with individual proteins to determine which proteins contributed most to protection. Using each of the proteins contained within the combination plus an additional protein that was highly stimulatory in the splenocyte assay (Figure 2; SP 0862), we immunized mice intranasally with vaccines comprised of single proteins and CT. As shown in Figure 4, SP2070 was highly protective (over 2 log reduction in median density of colonization, *p = *0.005 vs. CT control). Additionally, SP1534 and SP0862 conferred statistically significant reduction in colonization compared with CT-immunized controls (over 1 log reduction in median density of colonization). Animals immunized with SP2092 were not protected, as predicted by the low IL-17A elicited by this protein in the splenocytes stimulation assay.

## Discussion

We present a sequential method that we used to identify pneumococcal T cell antigens that, when administered as mucosal vaccines with CT, elicit IL-17A responses and provide protection against pneumococcal colonization. In particular, SP2070 was highly protective in our model and SP0862 and SP1534 both significantly reduced colonization.

In an effort to overcome the limitations of PCV-based approaches, many investigators have explored several serotype-independent approaches [5]. A protein subunit vaccine comprised of antigens that are well expressed and conserved across the majority of infecting serotypes is one such approach. Different methods have been applied to identify immunodominant pneumococcal antigens, such as a genomic approach to identify protective proteins that express a secretion signal [15], identification of protective proteins from human antibody screening using genomic scale antigenic fingerprinting [16] and proteomic screening to identify T_H_17 dependent antigens [14]. Here, instead of using a genomic-scale approach, we used a multi-step biochemical approach; 1) preparative SDS PAGE to size separate fractions of the immunogenic soluble compartment of a pneumococcal whole cell antigen 2) screening of fractions for IL-17 stimulatory properties *in vitro* 3) mass spectrometry of stimulatory fractions to identify comprising proteins and 4) identification of protective T_H_-17 antigens *in vivo*. A similar approach has been used to identify IFN-γ stimulatory antigens in mycobacterium tuberculosis [17] and might be applicable to other pathogens for which T-cell mediated immunity to infection is thought to play an important role.

There are several advantages to such a non-genomic approach to immunodominant antigen identification; namely, this method eliminates the need to generate a comprehensive antigenic expression library. The method used here can be completed relatively quickly and identifies a small number of immunodominant bands each comprised of several proteins which can be individually screened either *in vitro* or *in vivo* for immunogenic and protective potential, respectively. A limitation of this approach as it was implemented here was that the starting material (WCCsup) represents only the soluble compartment of pneumococcal cellular growth, and does not address the potential of proteins in the nonsoluble compartment. Additionally, we did not evaluate different concentrations of the individual proteins used in splenocyte stimulations so it is conceivable that the optimal stimulatory potential of any individual protein was not evaluated. However, as all of the identified proteins were within a relatively close molecular weight range (approximately 20–60 kDa) the molar amount represented in each stimulation was not drastically different.

We recognize that nasal mucosal cells such as nasal associated lymphoid tissue (NALT) or draining lymph nodes may represent more relevant immune cells than the splenocytes that were examined in the present study. Indeed, we have previously documented the presence of IL-17A response from nasal mucosal cells of WCV immunized mice [10]. However, the use of nasal mucosal tissue for screening of potential immunogens poses several challenges. First, evaluation of nasal mucosal lymphocyte responses to multiple antigens from individual mice is not feasible, as the numbers of cells that we can obtain from such tissue are too low; pooling of cells from multiple animals is required. To be able to perform the number of stimulations carried out with each of the fractions and proteins in this study, a prohibitively large number of mice would have been required. Furthermore, IL-17A responses from nasal mucosal tissue are almost 2-log lower than from splenocytes [10], which may reduce our ability to discriminate between stimulatory and non-stimulatory fractions and proteins. For these reasons, we favored the use of splenocytes for IL-17A screening assays.

Amongst the twelve antigens identified, one antigen SP1572 (also known as PppA), has been shown to be protective against colonization and sepsis and was therefore not studied in this paper [18], [19]. When the four most IL-17A immunogenic antigens were each separately used as mucosal vaccines with CT, three of them conferred significant reduction in colonization compared with CT-immunized animals. Of these, SP2070 has been identified from the cell wall fraction as an immunodominant target for age-dependent antibody responses in children's sera but has not been tested for protection against invasive disease [20]. When we compared our list with the top T_H_17 antigens that our group in collaboration with Genocea Biosciences and PATH identified following proteomic screening [14], there was no overlap. Because of differences between the two screens, it is perhaps not surprising that the strongest hits in the more comprehensive screen may not be the same as those identified here. The previous proteomic screen identified T_H_17 antigens from the pneumococcal proteome that stimulated robust IL-17A from CD4+ T cells of mice immunized with a pneumococcal whole cell vaccine; in contrast, here we identified T_H_17 antigens from a single predominant protein band within an immunogenic fraction of the chloroform-soluble fraction of a whole cell preparation. There are many possible reasons why the results of the two screens differ, such as differences in the proteins present in the chloroform-soluble fraction vs. the most abundant proteins in the whole cell vaccine. Additionally, the comprehensive screen relied on complex statistical analyses for identification of a “hit," criteria that were not required for the screen presented here.

Since we are aiming to develop a pneumococcal vaccine to provide serotype-independent immunity, it will be important to test the efficacy of any of the protective proteins against strains of diverse serotypes and/or multilocus sequence types (MLST). From the identified panel of antigens, (initially consisting of 37 proteins) we used several bioinformatic filters to further narrow the panel, including the requirement for high sequence homology across all sequenced pneumococcal strains. Of the 12 identified antigens, all but 2 had ≥98% homology at the amino acid level with the 22 available sequenced strains; SP 1572 and SP2092 had 89% and 95% homology, respectively. Plans to test the ability of these proteins to protect against strains of different serotypes and MLST are underway.

Intranasal immunization with these antigens provides protection against colonization, but such a route requires the use of an adjuvant such as CT, which is not appropriate for human use. In our experience, subcutaneous immunization of purified proteins with aluminum hydroxide did not induce IL-17A production unless proteins were introduced in a fusion conjugate construct [21]. It is conceivable that development of a pneumococcal protein subunit vaccine would contain several antigens and/or be formulated with different or novel adjuvants, or perhaps as a fusion-conjugate [21] to improve immunogenicity and facilitate parenteral administration. Fusion conjugates comprised of the proteins identified here with either pneumococcal cell wall polysaccharide or *Salmonella typhi* Vi polysaccharide to provide protection against pneumococcal and/or *S. typhi* diseases are currently under investigation within our laboratory [22].

In conclusion, using this sequential method of size separation of immunodominant fractions of the soluble compartment of a pneumococcal whole cell preparation and mass spectrometry to identify the comprising proteins, three out of the four top IL-17A stimulatory proteins tested in a colonization model were found to be protective while the lowest IL-17A stimulatory protein conferred no protection. The proteins identified with this method may represent promising candidates for inclusion in a protein-based pneumococcal vaccine.
